# Emotional Arousal‐Induced Episodic Memory Benefits Are Attenuated in Autism Spectrum Disorders, Especially in Older Age

**DOI:** 10.1002/aur.70083

**Published:** 2025-07-14

**Authors:** Sidni A. Justus, Emily Hutson, Justin Summe, Audrey Duarte

**Affiliations:** ^1^ Department of Psychological Science Kennesaw State University Kennesaw Georgia USA; ^2^ School of Psychology Georgia Institute of Technology Atlanta Georgia USA; ^3^ Department of Psychology University of Texas at Austin Austin Texas USA

**Keywords:** aging, arousal‐enhanced memory, ASD, autism, depression, emotional memory

## Abstract

Autism Spectrum Disorder (ASD) is a common neurodevelopmental disorder associated with episodic memory impairment. Although emotional factors such as arousal, as well as age and depression symptoms, are known to influence episodic memory in neurotypical (NT) populations, how these factors affect memory processes in ASD, which is associated with a higher prevalence of depression, remains unclear. In this large‐scale online study, 326 adults (ages 18–67) with or without ASD (*n* = 163 per group) and varying levels of depressive symptoms rated their experienced arousal of positive, negative, and neutral images and performed a recognition task 48 h later. Adults with ASD reported lower arousal for positive images and exhibited reduced arousal‐enhanced memory benefits for both positive and negative images compared to NT adults, independent of depression severity. Age further exacerbated this reduced arousal memory benefit in the ASD group, specifically for positive stimuli. These findings underscore the role of atypical emotional arousal in ASD on episodic memory, with age‐related declines suggesting accelerated vulnerability in positive memory retention.

**Summary**

- Adults with Autism Spectrum Disorder (ASD) often experience emotions differently, which can affect how well they remember important events. In this study, we found that adults with ASD had weaker memory for emotionally charged images, especially positive ones, and that these challenges increased with age, regardless of depression symptoms. These findings highlight the importance of understanding how emotional and age‐related factors contribute to memory challenges in ASD.

Episodic memories, or memories for personally experienced events, include a variety of contextual details such as time, place, as well as our emotional state (Allen et al. 2008; Tulving 1985). Studies of neurotypical (NT) adults suggest that emotionally salient events are often remembered better than neutral events (LaBar and Cabeza 2006 for review). Emotional memory is evolutionarily adaptive (e.g., memory of a dangerous encounter helping us avoid similar dangers in the future; Eaton and Anderson 2018) and has been linked to overall life satisfaction and well‐being as we age (Bohlmeijer et al. 2007 for meta‐analysis). These adaptive benefits underscore the importance of understanding emotional memory across the lifespan in populations with unique cognitive and socioemotional characteristics such as Autism Spectrum Disorder (ASD; American Psychiatric Association 2022) – one of the most common neurodevelopmental disorders (Salari et al. 2022 for review).

ASD is associated with deficits in episodic memory (see Desaunay et al. 2020; Griffin et al. 2022 for meta‐analyses), but research examining emotional memory in ASD is limited and yields mixed results (e.g., Beversdorf et al. 1998; Gaigg and Bowler 2008; Maras et al. 2012; South et al. 2008). Emotional stimuli could be characterized by level of arousal, ranging from calm to highly stimulating (Russell 1980; Barrett et al. 2007). Arousal, or the intensity of the emotional response, plays a significant role in allocation of attention and can affect both encoding and retrieval of emotional memories (LaBar and Cabeza 2006; Kensinger and Ford 2020, for review). In NT individuals, a well‐established arousal‐enhanced memory benefit (Cahill and McGaugh 1995; Kensinger and Corkin 2003; Mather and Sutherland 2011) suggests that highly arousing stimuli are better remembered than less arousing stimuli. This phenomenon has been widely supported by both behavioral and neural evidence in the NT literature (Kensinger and Ford 2020; Hamann 2001 for review). However, ASD is characterized by socioemotional differences (Gaigg 2012 for review; Uljarevic and Hamilton 2013 for meta‐analysis), including altered emotional arousal ranging from hypo‐ or hyper‐arousal to various stimuli, which can impact how emotional and social information is processed. For example, ASD‐related arousal differences may manifest as reduced reactions in emotionally charged situations (e.g., flat affect or lack of excitement during a celebration) or heightened reactions in neutral scenarios (e.g., feeling overwhelmed in routine environments such as a grocery store). These patterns can meaningfully impact day‐to‐day functioning by shaping how individuals with ASD experience and navigate emotional and sensory information in real‐world contexts. Further, atypical arousal patterns in either direction could affect attention during encoding, leading to deficits in memory for emotionally salient events in ASD. For example, blunted arousal may limit attentional engagement during encoding, reducing the memory strength (Bradley et al. 2012). Conversely, hyper‐arousal could overwhelm attentional resources (to the point of distraction), impairing both encoding and later retrieval (e.g., Strength and Vulnerability Integration [SAVI] model, Charles 2010). These patterns underscore the importance of examining arousal's role in encoding to determine how memory deficits may arise from atypical arousal patterns in ASD.

Despite findings that arousal impacts memory performance in NT adults, limited ASD emotional memory literature has produced inconsistent findings regarding arousal‐enhanced memory in this population. Beversdorf et al. (1998) observed a reduced memory benefit for negative compared to neutral sentences in adults with ASD compared to NT adults and suggested this may be due to differences in arousal processing, though arousal levels were not directly measured. In contrast, South et al. (2008) found no group differences (adolescents and adults with ASD vs. NT) in memory performance across arousal categories (high, neutral, low), using stimuli with arousal levels defined by norms in NT adults. The two studies (Maras et al. 2012; Gaigg and Bowler 2008) that directly measured participants' physiological (i.e., galvanic skin response, heart rate) and subjective arousal to stimuli produced similarly mixed results. Gaigg and Bowler (2008) found that arousal‐enhanced memory benefits in ASD were only evident in immediate but not delayed recall, whereas NT showed consistent benefits even after 1‐h and 1‐day delays. Conversely, Maras et al. (2012) found no group differences in immediate nor delayed memory performance. Together, these findings suggest that emotional arousal may not reliably enhance memory of adults with ASD as it does in NT adults, particularly when memory retrieval is delayed. Further, while subjective arousal ratings were collected in both Gaigg and Bowler (2008) and Maras et al. (2012), they were not used to categorize arousal of the stimuli, making it unclear whether individual differences in arousal impacted memory performance.

Further, few existing studies have examined whether arousal‐enhanced memory benefits apply similarly across valence, which ranges from positive to negative (Russell 1980; Barrett et al. 2007). Although research in NT adults suggests that memory can be enhanced for both positive and negative stimuli (e.g., Ochsner 2000), studies of arousal and valence effects in ASD are limited and have been noted as a future research direction (Maras et al. 2012). Gaigg's (2012) review notes that ASD may involve valence‐dependent reactions in social situations (e.g., heightened responses to negative but blunted responses to positive social interactions), which further underscores the need for exploration of valence in emotional memory. Prior ASD studies often used either solely negative stimuli (Beversdorf et al. 1998; Maras et al. 2012), limiting insights into positive memory in individuals with ASD, or collapsed positive and negative stimuli into one emotionally arousing category (Gaigg and Bowler 2008) which may obscure valence‐specific effects. To our knowledge, only two ASD studies (Deruelle et al. 2008; South et al. 2008) have assessed memory for positive and negative arousal separately, with mixed findings and notable limitations. For example, Deruelle et al. (2008) included only a few stimuli (i.e., 6 of each positive, negative and neutral images) and relied on NT‐normed arousal ratings, which may lack generalizability given that individuals with ASD show atypical self‐reported arousal when rating emotional stimuli compared to NT controls (Bölte et al. 2008). Similarly, South et al. (2008) used pre‐determined arousal and valence categorizations to assess group differences in affective memory for 40 words. Further, the results from these two studies are inconclusive. Deruelle et al. (2008) found that ASD remembered emotional (both positive and negative) and neutral images similarly while NT showed the expected emotional enhancement effect (negative > positive and neutral). South et al. (2008) found no significant group differences in memory effects based on valence (negative > neutral > positive for all) or arousal (high > neutral > low for all). In the present study, we examine subjective arousal ratings and their effects on memory for both positive and negative relative to neutral stimuli, providing a more nuanced understanding of both valence and arousal effects on emotional memory in ASD.

The present study further addresses two important moderating variables, depression and age, which have not been consistently examined in prior ASD emotional memory research. Studies of NT populations show that depression, a common comorbidity in ASD (Hossain et al. 2020 for review) can impair episodic memory performance, especially for neutral and positive events, across the adult lifespan in NT adults (James et al. 2021 for meta‐analysis). Specifically, depression can influence memory by attenuating reactivity to positive stimuli (i.e., positive attenuation hypothesis; Clark et al. 1994) and exaggerating reactivity to negative stimuli (i.e., negative potentiation hypothesis; Beck 1967). Given that individuals with ASD are four times more likely to experience depression in their lifetime than NT peers (Hudson et al. 2019) and often self‐report elevated depression symptoms, even without a formal diagnosis (Gotham et al. 2015), it is crucial to assess whether depression symptoms influence emotional memory performance in this population. Only two existing studies of emotional memory in ASD (Deruelle et al. 2008; Gaigg and Bowler 2008) explicitly acknowledged excluding participants with comorbid psychiatric or neurological conditions, but neither measured depression symptomatology as a covariate of interest. Thus, it is unclear whether altered valence and/or arousal memory biases in ASD are related to the diagnosis, per se, or to comorbid depression. In the present study, we control for depression, making it possible to separate ASD‐specific effects on memory from effects of depression, and enhancing relevance to the broader ASD population.

Finally, the present study includes a wide age range, allowing for exploration of age effects on emotional memory in ASD. While a recent NT study from our lab suggests age‐related declines in arousal‐enhanced memory (Lee et al. 2023), the question of whether accelerated cognitive aging (i.e., earlier declines in arousal‐enhanced memory) may occur in ASD remains debated (Geurts and Vissers 2012; Torenvliet et al. 2023). Existing emotional memory studies in ASD have largely overlooked the impact of age as most only report matching on chronological age (e.g., Beversdorf et al. 1998; Gaigg and Bowler 2008; Maras et al. 2012) rather than exploring age as a covariate. The two ASD studies exploring age as a covariate (Deruelle et al. 2008; South et al. 2008) reported no significant age effects. However, small sample sizes (15 per group in Deruelle, 37 per group in South) and a restricted age range (17–55 years in Deruelle, 11–38 years in South) may have limited the ability to reliably explore individual differences.

In sum, although emotional factors such as arousal, age, and depression symptoms are known to influence episodic memory in NT populations, their respective and/or synergistic effects on memory processes in adults with ASD remain largely unexplored, particularly given the higher prevalence of depression in this population. To our knowledge, this is the first large‐scale study to explore subjective arousal's influence on memory performance in individuals with and without ASD. Participants rated arousal for positive, neutral, and negative images during encoding and performed a recognition memory task two days later. Within each valence condition (positive and negative), we specifically explored the influence of subjective arousal on memory enhancement. We hypothesized that adults with ASD may show reduced arousal‐related memory benefits compared to NT adults, based on the tendency observed in some studies for atypical (either hypo‐ or hyper‐) emotional arousal to various stimuli. Given the higher prevalence of depression in ASD, we also hypothesized that depression would independently contribute to memory outcomes, potentially compounding the effects of ASD diagnosis on arousal‐enhanced memory performance. By including the level of depression severity in our analyses, predicted to be higher in ASD than NT adults, we aimed to disambiguate its association with the memory outcomes from that of ASD diagnosis. Additionally, considering that aging has been linked to reductions in arousal‐enhanced memory in NT adults, we explored age as another potential moderator. Specifically, we examined whether aging may exacerbate memory performance differences in ASD. By examining arousal, depression, and age in a single study, we aim to clarify the distinct roles of these factors in ASD‐related emotional memory differences, with potential implications for understanding broader social and cognitive challenges observed in this population.

## 1 Method

### 1.1 Participants

All participants were recruited for the study through Prolific, an online data collection platform (www.prolific.com). Inclusion criteria included that all participants were native English‐speaking adults located in the United States, between ages 18–80 years, with normal or corrected‐to‐normal vision. ASD diagnosis was evaluated through multiple means (see Autism assessment). Of the 195 recruited participants with ASD, 164 completed the entire (i.e., both the encoding and retrieval sessions) study procedure. One additional participant was excluded from analysis for poor quality responses (i.e., 10 + % of data missing, or identical responses given for the majority of trials). The remaining 163 ASD participants (75 males, 61 females, 27 genderqueer or other; age: range 18–67 years, M_*age*_ = 29.94, SD_*age*_ = 9.92; education: 19 graduate degree, 44 four‐year/Bachelor's degree, 18 two‐year/Associate's degree, 51 some college, 30 high school/GED, 1 less than high school) were then best‐available matched based on age, gender, and education to NT participants (74 males, 75 females, 14 genderqueer or other; age: range 18–67 years, M_*age*_ = 30.10, SD_*age*_ = 9.78; education: 17 graduate degree, 56 four‐year/Bachelor's degree, 18 two‐year/Associate's degree, 46 some college, 24 high school/GED, 1 less than high school) from an existing dataset (*n* = 585) collected by our lab that utilized an identical experimental paradigm. A subset of this NT sample data was included in a prior publication (Lee et al. 2023). Informed consent was obtained electronically, and participants were compensated at a rate of $10/h. The study was approved by the Georgia Institute of Technology Institutional Review Board.

### 1.2 Materials

#### 1.2.1 Autism Assessment

ASD diagnosis is usually confirmed for in‐person research via a clinical interview using the Autism Diagnostic Observation Schedule‐2 (ADOS‐2; Lord et al. 2012). Due to the online nature of this study, an ADOS‐2 could not be completed. Instead, participants in the ASD group all first reported a clinical diagnosis of ASD received either as a child or as an adult in their Prolific profile. Second, participants had to re‐indicate such diagnosis in the health questionnaire embedded in the task (see Supporting Information). Participants were further asked to provide information about the age of diagnosis, diagnosis received, and the name or type of diagnosing medical professional. Previous online studies (e.g., Baron‐Cohen et al. 2015; Atherton et al. 2022; McDonnell et al. 2024) have utilized similar methods to verify self‐reported diagnosis in autistic adults. Although Daniels et al. (2012) focused on parent‐report within a clinical registry, their findings support the broader notion that the ability to provide detailed diagnostic information is highly correlated with confirmed clinical diagnosis. In this study, inclusion criteria were similar to Baron‐Cohen et al. (2015) in that participants had to report a clinical diagnosis of autism as would be in accordance with DSM‐IV (i.e., autism spectrum condition or any pervasive developmental disorder), DSM‐5 (autism spectrum disorder), or ICD‐10 (any pervasive developmental disorder) from a recognized developmental specialist such as a psychiatrist or clinical psychologist. Finally, participants with ASD were asked to complete self‐report symptomatology questionnaires, the Autism Quotient (AQ; Baron‐Cohen et al. 2001) and the Social Responsiveness Scale‐2 (SRS‐2; Constantino and Gruber 2012) to measure current symptom severity. In this study, these questionnaires were not used to exclude ASD participants, but rather to characterize current symptom severity. Neurotypical participants in this study did not self‐endorse ASD diagnosis in their Prolific participant profiles or our eligibility questions and, therefore, were not asked to complete the autism‐specific questionnaires (i.e., AQ, SRS‐2).

The AQ contains 50 items (e.g., I prefer to do things with others rather than on my own”) that participants indicate how well each item describes themselves using a Likert scale from (“1 = definitely disagree” to 4 = definitely agree”). The original Baron‐Cohen et al. (2001) dichotomous method collapsed scores into 0 (definitely/slightly disagree) and 1 (definitely/slightly disagree) with a max score of 50 and scores of 31+ indicating clinical significance. In the present study, the entire 4‐point scale was used to score AQ scores, which has been shown to increase reliability and validity and retain response detail and variability (Stevenson and Hart 2017). Total scores ranged from 50 to 200 with higher scores indicating increased level of autistic traits.

The SRS‐2 contains 65 items measuring five sub‐scales: social awareness (e.g., “I am usually aware of how others are feeling”), social cognition (e.g., “I recognize when something is unfair”), social communication (e.g., “I am able to communicate my feelings to others”), social motivation (e.g., “I am much more uncomfortable in social situations than when I am by myself”), and restricted interests or behaviors (e.g., “I have more difficulty than others with changes in my routine”). Participants are asked to respond how well each statement describes their behavior over the last six months using a Likert scale from “1 = not true” to “4 = almost always true.” Raw scores for each of the five domains and total score are converted into normed T‐scores for interpretation. T‐scores range from 30 to 90 with higher scores indicating greater levels of autistic traits.

#### 1.2.2 Depression

The Center for Epidemiological Studies Depression Scale Revised (CESD‐R; Eaton et al. 2004) was used to measure depression symptomatology. The CESD‐R contains 20 items measuring nine sub‐subscales (2–3 items each) of depression: sadness (e.g., “I could not shake off the blues”), loss of interest (e.g., “Nothing made me happy”), appetite (e.g., “My appetite was poor”), sleep (e.g., “My sleep was restless”), thought/concentration (e.g., “I had trouble keeping my mind on what I was doing”), guilt (e.g., “I felt like a bad person”), fatigue (e.g., “I could not get going”), movement (e.g., “I felt like I was moving too slowly”), and suicidal ideation (e.g., “I wanted to hurt myself”). Participants are asked to respond to how often they have felt or behaved like each of the items over the last week or more on a Likert scale from “0 = not at all or less than one day” to “4 = nearly every day for two weeks.” Total scores are summed from all 20 items and range from 0 to 60. Higher scores indicate increased depression symptomatology (scores < 16 = no clinical significance).

#### 1.2.3 Stimuli

The emotional memory task for this study utilized 396 images that were selected from the International Affective Picture System (IAPS; Lang et al. 1997) and the Nencki Affective Picture System (NAPS; Marchewka et al. 2014) image banks. The images were categorized into 132 positive (M_valence_ = 7.12 ± 0.39, M_arousal_ = 5.47 ± 0.61), 132 neutral (M_valence_ = 5.62 ± 0.44, M_arousal_ = 4.13 ± 0.72), and 132 negative (M_valence_ = 3.55 ± 0.83, M_arousal_ = 5.55 ± 0.44) photos, according to published norms for arousal and valence. The positive images were matched in arousal ratings to the negative images (t(262) = 1.24, *p* = 0.215), but were both significantly more arousing than the neutral images (*t*'s > 16.34, *p*'s < 0.001). Eighteen of the 396 images were presented during practice. Two hundred and fifty‐two of the images (84 of each valence type) were included in the encoding phase. An additional 126 images (42 of each valence type) were included as “new” stimuli during retrieval. Old and new item assignment was counterbalanced across participants.

### 1.3 Procedure

The present study featured questionnaires administered via Qualtrics (Qualtrics, Provo, UT, https://www.qualtrics.com) and an emotional memory task created using PsychoPy (Peirce et al. 2019) and hosted for online data collection via Pavlovia (Ilixa Ltd., Nottinghamshire, UK, https://pavlovia.org). Participants were informed of study procedures on the Prolific advertisement, and electronic consent was obtained via Qualtrics.

Participants completed two online sessions, 48‐h apart, to minimize ceiling effects and to elicit greater emotion‐related enhancement of memory performance (Yonelinas and Ritchey 2015). Participants performed the encoding task in the first session and the retrieval task in the second session. During the first session, participants also completed the health questionnaire and battery of other self‐report screeners described above to assess autism (AQ and SRS) and depression (CESD‐R) symptomatology. During the second session, participants completed a demographics questionnaire and additional measures that were part of a larger research project (e.g., Global Physical Activity Questionnaire [GPAQ]; Armstrong and Bull 2006). Measures not directly related to the present research questions are not included in this paper.

**FIGURE 1:**
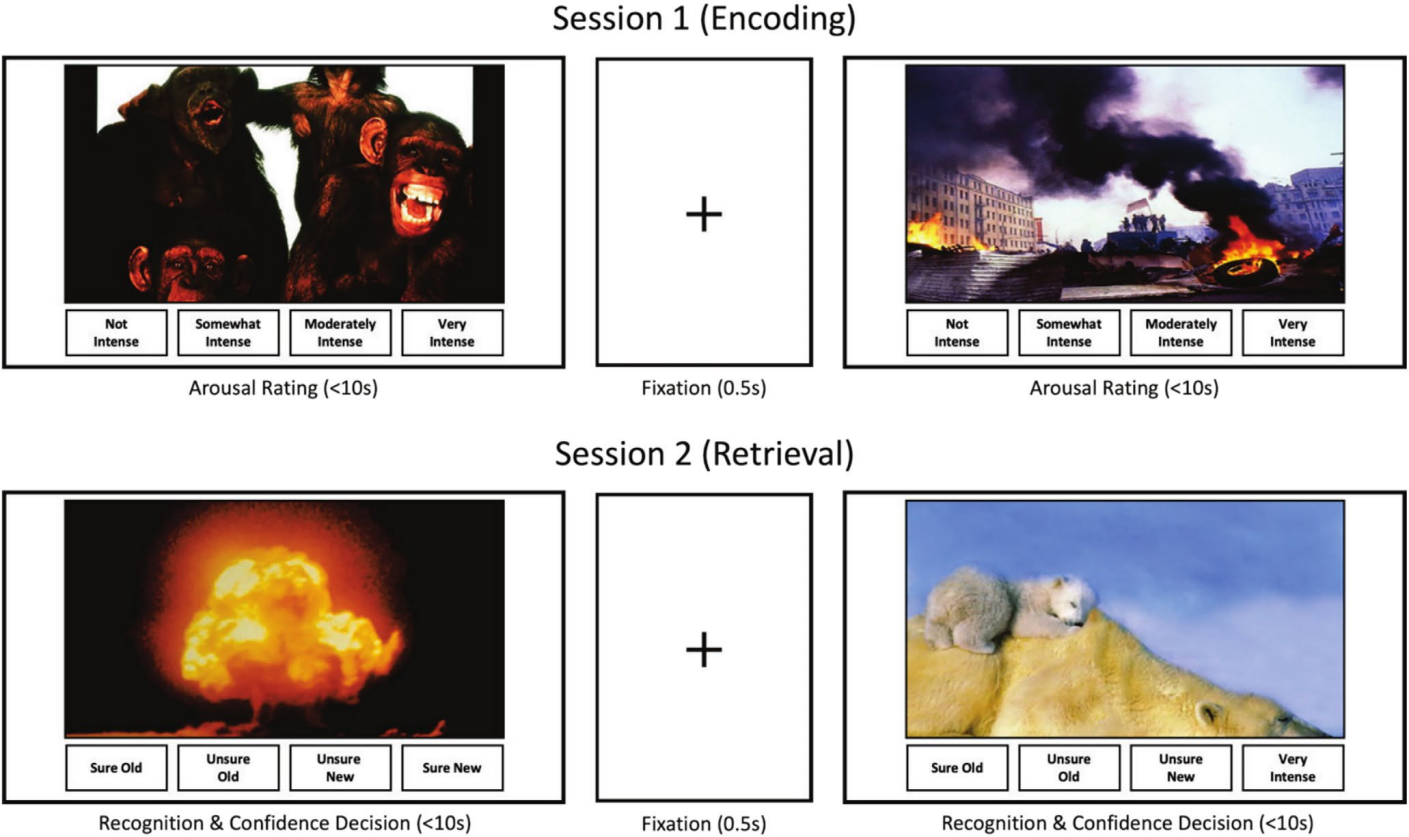
Experimental design.

Figure 1 illustrates both session 1 (encoding task) and session 2 (retrieval task) structure and timing. The encoding task (252 trials) was split into four blocks of 63 trials. Each block contained an equal number (*n =* 21) of positive, negative, and neutral images, presented in pseudorandom order with no more than four consecutive stimuli of the same valence. Participants were instructed to rate their arousal in response to each image on a four‐point scale: 1 = not intense, 2 = somewhat intense, 3 = moderately intense, 4 = very intense. Participants were given a maximum of 10s to respond and 500 ms separated each trial. Prior to the emotional intensity rating task, participants viewed a brief (3.5‐min) instructional video. They were informed that they would see a series of positive, neutral, and negative images and should rate how strong of an emotional reaction they personally experienced to each image using the 4‐point scale (1 = not intense, 4 = very intense) shown in Figure 1. Visual and narrated instructions emphasized that there were no right or wrong answers and responses should reflect their own emotional experience. The video also reminded participants that once a response was made, they would automatically proceed to the next trial with no opportunity to change their answer. The video then walked participants through a series of examples to reinforce subjectivity (e.g., someone might rate a photo of a skier as very intense if they enjoy skiing, whereas others may feel neutral). Participants then proceeded to the encoding task, where they completed 18 practice trials before moving on to the experimental blocks.

Approximately 48 h later, participants completed the retrieval task (378 trials) containing all 252 encoding images and 126 new images. The retrieval task was split into six blocks of 63 trials (42 old, 21 new images per block). Each block contained an equal number of positive, negative, and neutral images presented in a pseudorandom order. Participants were instructed to judge whether each image was old or new and to indicate confidence associated with their decision using a four‐point scale: 1 = sure old, 2 = unsure old, 3 = unsure new, 4 = sure new. Participants were given a maximum of 10 s to respond, and 500 ms separated each trial. Prior to the memory task, participants again viewed a brief (3.5‐min) instructional video. They were informed that they would complete a memory task for the images seen in the first part of the experiment completed a few days ago. They would see an image and should use their mouse to indicate whether the image is old or new and their confidence in their decision by selecting one of four response options (Sure Old, Unsure Old, Unsure New, or Sure New) shown in Figure 1. The use of the scale point was described (e.g., if you are certain you saw the image in the first part of the study, you would select sure old). Instructions emphasized that participants should respond based on their memory of the first part of the experiment. Participants were again reminded that once a response was made, they would automatically proceed to the next trial with no opportunity to change their answer. This video also included sample trials to illustrate how to use the scale. Participants then proceeded to the memory task, where they completed 18 practice trials before moving on to the experimental blocks.

### 1.4 Analyses

Statistical analyses were performed using SPSS (IBM Corp 2022). Individual average subjective arousal was calculated for positive, neutral, and negative images. Memory performance was estimated separately for positive, neutral, and negative images using *d'prime* discrimination index (*d*’ = *z*(hit rate) – *z*(false alarm rate)). Invalid trials (i.e., trials with responses < 200 ms) were excluded from analysis. For positive and negative images, *d’* based on subjective arousal was further calculated such that high arousal collapsed ‘very intense’ and ‘moderately intense’ ratings and low arousal collapsed across ‘somewhat intense’ and ‘not intense’ ratings. Neutral images are inherently low in arousal and were not further divided into high/low categories. Participants who did not have at least five hits in either high or low arousal categories were excluded in the calculation of *d’* for that category. This resulted in the following sample sizes: Positive Low Intensity *d*' (ASD = 157, NT = 161), Positive High Intensity *d*' (ASD = 131, NT = 131), Negative Low Intensity *d*' (ASD = 159, NT = 161), Negative High Intensity *d*' (ASD = 147, NT = 161).

For all analyses, we evaluated whether conditions were met for applying parametric tests by inspecting box plots for significant outliers and testing for violations of normality (Kolmogorov–Smirnov test *p* < 0.05) and homoscedasticity (Levene's test *p* < 0.05). For instances of violated normality, we re‐ran analyses after removing outliers. In all such cases, no changes in statistical significance were found for the results presented below. Thus, we reported parametric results from full data for all measures. Correlation analyses were used to illustrate general relationships among predictor and outcome variables for each group separately. We grand mean centered all continuous predictor variables prior to analysis to reduce multicollinearity and aid interpretation of interactions. Hierarchical multiple regressions were conducted to investigate the influence of group (NT = 0, ASD = 1), age, and depression level on subjective arousal and memory performance. Group, depression, and age were entered as predictors in the first block. Group × Age and Group × Depression interactions were entered in the second block. The outcome variables of interest were subjective arousal ratings, memory preferences (based on valence), and arousal‐enhanced memory (i.e., high arousal *d*′ – low arousal *d*′). Analysis of variance (ANOVA) and analysis of covariance (ANCOVA) effect sizes were calculated using partial eta‐squared ($\eta_{p}^{2}$), where $\eta_{p}^{2}$ = 0.01, $\eta_{p}^{2}$ = 0.06, $\eta_{p}^{2}$ = 0.14 is considered small, moderate, or large effect size, respectively (Cohen 2013). Greenhouse‐Geiser corrections were applied as needed when the assumption of sphericity was violated. T‐test effect sizes were calculated using *Cohen's d*, where d = 0.2, d = 0.5, d = 0.8 is considered small, medium, or large effect size, respectively (Cohen 2013).

## 2 Results

### 2.1 Demographics and Neuropsychological Tests

Age and neuropsychological test results are shown in Table 1. Adults with ASD reported greater depression symptomatology [*t*(324) = 4.14, *p* < 0.001, Cohen's *d =* 0.46]. Separate correlation analyses were conducted as preliminary checks to evaluate the relationships between predictor variables (i.e., age, depression) within each group. Given the higher prevalence of depression in ASD, these correlations allow us to determine whether age, depression, and ASD symptoms show underlying associations that could impact the hierarchical regression analyses. Age was negatively correlated with depression in adults with ASD (*r* = −0.182, *p* = 0.02), but this relationship was not significant for the NT group (*r* = −0.127, *p* = 0.105). We also ran correlation analysis for the ASD group to determine whether self‐reported ASD symptoms related to predictors of interest (age, depression). ASD symptoms were positively correlated with age for the AQ (*r* = 0.167, *p* = 0.033) but not the SRS‐2 (*r* = −0.014, *p* = 0.860). ASD symptoms were also positively correlated with depression for both the AQ (*r* = 0.158, *p* = 0.043) and SRS‐2 (*r* = 0.412, *p* < 0.001).

**TABLE 1:** Participant demographics.

Measure	NT		ASD	
	Range	M (SD)	Range	M (SD)
Age^a^	18–67	30.10 (9.78)	18–67	29.94 (9.92)
CESD‐R^b^	0–55	18.00 (14.11)*	0–60	24.74 (15.28)*
AQ^c^	—	—	77–189	138.52 (20.45)
SRS‐2 (*T*‐score)^d^				
Social awareness	—	—	41–90	61.74 (9.39)
Social cognition	—	—	39–90	64.93 (9.78)
Social communication	—	—	37–90	68.57 (11.36)
Social motivation	—	—	37–87	67.65 (9.27)
Restricted interests/behavior	—	—	40–90	71.25 (12.42)
Total	—	—	37–90	69.55 (10.46)

^a^ Indicates variable used for matching.

^b^ Center for Epidemiological Studies Depression Scale Revised.

^c^ Autism Quotient.

^d^ Social Responsiveness Scale‐2, adult form self‐report.

* Indicates significant group difference (*p* < 0.05).

### 2.2 Subjective Arousal Ratings

Average group subjective arousal ratings for each valence category are shown in Table 2. To test the prediction that subjective arousal ratings would vary between groups (NT vs. ASD) and across valence categories (Positive, Neutral, Negative), we conducted a Group × Valence ANOVA. Results revealed a main effect of Valence, [*F*(1.81, 585.33) = 848.42, *p* < 0.001, $\eta_{p}^{2}$ = 0.73]. Across all participants, arousal ratings were highest for negative and lowest for neutral images (positive vs. neutral: *t*(325) = 23.69, *p* < 0.001, Cohen's *d* = 1.31; negative vs. neutral: *t*(325) = 38.71, *p* < 0.001, Cohen's *d* = 2.14; negative vs. positive: *t*(325) = 19.78, *p* < 0.001, Cohen's *d* = 1.10). There was no main effect of Group, [*F*(1, 324) = 0.001, *p* = 0.978, $\eta_{p}^{2}$ = 0.00], however a significant Group*Valence interaction, [*F*(1.81, 585.33) = 5.67, *p* = 0.005, $\eta_{p}^{2}$ = 0.02] was revealed. Follow‐up analyses were conducted to explore the Group*Valence interaction. Independent samples t‐tests revealed no significant differences between NT and ASD groups in mean arousal ratings within each valence (*t*(324)'s < 1.44, *p*'s > 0.152, Cohen's *d*'s < 0.16). Separate ANOVAs for each group revealed a main effect of Valence in both groups, [NT: *F*(1.71, 276.17) = 389.75, *p* < 0.001, $\eta_{p}^{2}$ = 0.71; ASD: *F*(1.77, 287.12) = 479.16, *p* < 0.001, $\eta_{p}^{2}$ = 0.75]. Independent samples *t*‐tests on difference scores between valence were conducted to compare changes in arousal ratings between groups. Mean arousal differences for negative vs. positive images were larger for adults with ASD (M_*diff*_ = 0.57, SE = 0.03) compared to NT adults (M_*diff*_ = 0.42, SE = 0.04), *t*(324) = −3.01, *p* = 0.003, Cohen's *d* = 0.44, equal variances not assumed as Levene's test was significant. Conversely, for positive versus neutral images, mean differences were larger for NT adults (M_*diff*_ = 0.49, SE = 0.03) than for adults with ASD (M_*diff*_ = 0.39, S*E* = 0.03), *t*(324) = 2.85, *p* = 0.005, Cohen's *d* = 0.32. For negative vs. neutral images, there was no significant difference between adults with ASD (M_*diff*_ = 0.96, SE = 0.04) and NT adults (M_*diff*_ = 0.92, SE = 0.03), *t*(324) = −0.87, *p* = 0.385, Cohen's *d* = 0.10.

**TABLE 2:** Group subjective arousal ratings and memory discriminability (*d*′) as mean (standard deviation) split by valence.

	Positive	Neutral	Negative
Arousal rating			
NT	1.97 (0.55)	1.47 (0.40)	2.39 (0.45)
ASD	1.88 (0.53)	1.49 (0.40)	2.45 (0.55)
Overall *d*'			
NT	1.43 (0.66)	1.44 (0.74)	1.77 (0.76)
ASD	1.45 (0.68)	1.41 (0.77)	1.69 (0.73)
Low Arousal *d*'			
NT	1.39 (0.66)^†^	—	1.61 (0.75)^†^
ASD	1.37 (0.65)^†^	—	1.53 (0.78)^†^
High Arousal *d*'			
NT	1.66 (0.73)^†^	—	2.03 (0.82)^†^
ASD	1.49 (0.86)^†^	—	1.79 (0.79)^†^

*Note*: Entries are in the format: Mean (SD). *d'* = *z*(hit rate)—*z*(false alarm rate). Participants who had less than 5 valid trials in either the high or low arousal category were excluded in the calculation of *d*′ for that category ^†^ *n* = Positive Low Arousal *d*' (ASD = 157, NT = 161), Positive High Arousal *d*' (ASD = 131, NT = 131), Negative Low Arousal *d*' (ASD = 159, NT = 161), Negative High Arousal *d*' (ASD = 147, NT = 161).

To examine how age and depression might moderate subjective arousal ratings corrected for baseline arousal, we conducted separate hierarchical regressions (Table 3). Positive and negative subjective arousal were each baseline corrected by subtracting individuals' neutral intensity ratings prior to analysis. Results revealed that the previous finding that NT adults reported higher arousal ratings for positive (compared to neutral) images compared to adults with ASD was not moderated by age or depression symptoms. Additionally, older age was associated with increased arousal for positive images while higher symptoms of depression were associated with decreased arousal for positive images. There were no significant predictors of subjective arousal for negative compared to neutral images.

**TABLE 3:** Hierarchical multiple regression with age, group, and depression predicting positive and negative arousal.

	Model 1			Model 2			Model 3		
	*B*	SE *B*	*β*	*B*	SE *B*	*β*	*B*	SE *B*	*β*
Positive – neutral arousal									
Group	−0.078	0.037	−0.115*	−0.078	0.037	−0.115*	−0.078	0.037	−0.115*
Depression	−0.004	0.001	−0.178**	−0.004	0.002	−0.195*	−0.004	0.002	−0.193*
Age	0.005	0.002	0.148**	0.005	0.002	0.149**	0.006	0.003	0.162*
Group × Depression	—	—	—	0.001	0.002	0.022	0.001	0.003	0.019
Group × Age				—	—	—	−0.001	0.004	−0.019
*R* ^2^		0.076			0.073			0.071	
*F* for Δ*R* ^2^		9.915***			7.437			5.943	
Negative – neutral arousal									
Group	0.036	0.050	0.041	0.035	0.050	0.039	0.034	0.050	0.039
Depression	0.001	0.002	0.031	0.004	0.002	*0.127*	*0.004*	*0.002*	0.121
Age	−0.001	0.003	−0.027	−0.001	0.003	−0.029	−0.003	0.004	−0.073
Group × Depression	—	—	—	−0.005	0.003	−0.128	−0.005	0.003	−0.118
Group × Age				—	—	—	0.004	0.005	0.063
*R* ^2^		−0.005			−0.001			−0.002	
*F* for Δ*R* ^2^		0.457			0.946			0.881	

*Note*: *p < 0.05, **p < 0.01, ***p < 0.001. *R* ^2^ represents adjusted *R* ^2^. Group (0 = NT, 1 = ASD). *B*, unstandardized regression coefficient; SE *B*, standard error of the unstandardized coefficient; β, standardized regression coefficient.

### 2.3 Memory Performance

Average memory performance (*d'prime*) split by valence for each group is also shown in Table 2. To test the prediction that memory performance would differ between groups (NT vs. ASD) and across valence categories (Positive, Neutral, Negative), we conducted a Group × Valence ANOVA. Results revealed a main effect of Valence [*F*(2, 648) = 93.82, *p* < 0.001, $\eta_{p}^{2}$ = 0.23]. Across all participants, memory performance was highest for negative images and similar for positive and neutral images (positive vs. neutral: *t*(325) = 0.60, *p* = 0.552, Cohen's *d* = 0.03; negative vs. neutral: *t*(325) = 12.11, *p* < 0.001, Cohen's *d* = 0.67; negative vs. positive: *t*(325) = 11.82, *p* < 0.001, Cohen's *d* = 0.66). There was no main effect of Group [*F*(1, 324) = 0.16, *p* = 0.698, $\eta_{p}^{2}$ = 0.00], and no Group*Valence interaction [*F*(2, 648) = 2.18, *p* = 0.114, $\eta_{p}^{2}$ = 0.01].

**FIGURE 2:**
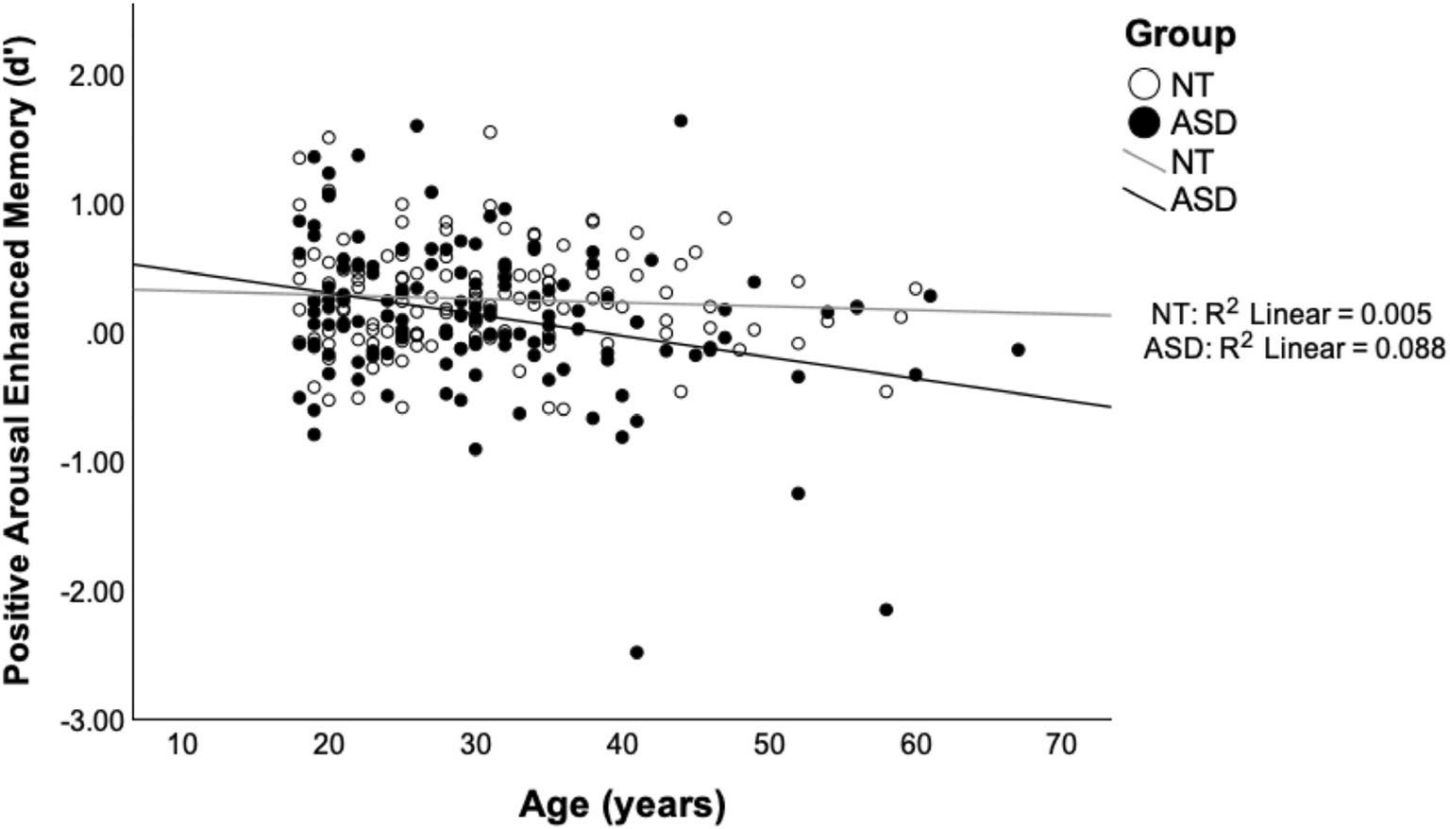
Age × Group interaction on arousal related memory benefit for positive stimuli.

To further explore the prediction of reduced arousal‐enhanced memory in ASD compared to NT, and to examine potential moderation of depression and/or age, we conducted hierarchical regression analyses (Table 4). Arousal related memory enhancement was calculated as the difference between high and low arousal memory (i.e., high arousal *d*′—low arousal *d*′). Results revealed that adults with ASD had lower arousal‐enhanced memory for positive items than did NT adults. Additionally, older age was also associated with a decreased arousal‐enhanced memory benefits for positive images. However, this main effect of age was further moderated by an interaction with Group in Model 3 (see Figure 2). Follow‐up regression analyses for each group separately revealed that age was a significant predictor of attenuated arousal memory benefits for adults with ASD only. Depression symptoms were not a significant predictor or moderator for positive arousal‐enhanced memory. Adults with ASD also had lower arousal‐enhanced memory benefit for negative items compared to NT. There were no other significant predictors identified for negative arousal‐enhanced memory.

**TABLE 4:** Hierarchical multiple regressions with age, group, and depression predicting arousal‐enhanced memory benefits (High Arousal *d*′ – Low Arousal *d*′).

	Model 1			Model 2			Model 3		
	*B*	SE *B*	*β*	*B*	SE *B*	*β*	*B*	SE *B*	*β*
Positive (High arousal *d*′ – Low arousal *d*′)									
Group	−0.129	0.063	−0.127*	−0.128	0.063	−0.126*	−0.116	0.063	−0.115
Depression	0.000	0.002	−0.014	−0.002	0.003	−0.071	−0.002	0.003	−0.059
Age	−0.010	0.003	−0.206***	−0.010	0.003	−0.203***	−0.003	0.004	−0.064
Group × Depression	—	—	—	0.004	0.004	0.077	0.002	0.004	0.049
Group × Age				—	—	—	−0.013	0.006	−0.193*
*R* ^2^		0.047			0.046			0.059	
*F* for Δ*R* ^2^		5.222**			4.089			4.255*	
Negative (High arousal *d*′ – Low arousal *d*′)									
Group	−0.121	0.053	−0.135*	−0.122	0.053	−0.136*	−0.120	0.053	−0.134*
Depression	0.002	0.002	0.064	0.001	0.003	0.030	0.001	0.003	0.037
Age	−0.004	0.003	−0.091	−0.004	0.003	−0.090	−0.002	0.004	−0.037
Group × Depression	—	—	—	0.002	0.004	0.047	0.001	0.004	0.034
Group × Age				—	—	—	−0.005	0.005	−0.077
*R* ^2^		0.018			0.016			0.016	
*F* for Δ*R* ^2^		2.894*			2.243			1.977	

*Note*: *p < 0.05, **p < 0.01, ***p < 0.001. *R* ^2^ represents adjusted *R* ^2^. Group (0 = NT, 1 = ASD) *B*, unstandardized regression coefficient; SE *B*, standard error of the unstandardized coefficient; β, standardized regression coefficient.

## 3 Discussion

Emotional memory impairments have important implications for understanding the socioemotional challenges in ASD, as poor recall of emotionally significant events, especially in social contexts, may limit adaptive interpersonal behavior. For instance, if individuals with ASD experience difficulty remembering emotionally charged social encounters, they may struggle to adjust their behavior based on past interactions, potentially compounding characteristic challenges in social relationships and broader emotional understanding associated with this population. In the present study, we aimed to extend previous work on emotional memory in ASD by investigating the effects of both valence and arousal influences on emotional memory within an adult lifespan sample of individuals with and without ASD.

Our results build on previous findings by demonstrating that adults with ASD show reductions in arousal‐enhanced memory benefits for both positive and negative stimuli, although the mechanisms underlying these reductions appear to vary by valence. Given prior research suggesting that atypical arousal responses may affect memory, we hypothesized that adults with ASD would exhibit diminished arousal‐enhanced memory for emotionally salient stimuli. Specifically, adults with ASD rated positive images as less arousing relative to neutral compared to NT adults, while no group differences in arousal ratings were found for negative images. This selective reduction in subjective arousal ratings for positive images supports our hypothesis and suggests that individuals with ASD may process or respond to positive emotional content differently. This finding is consistent with research on blunted emotional responses to positive social interactions despite typical or heightened responses to negative situations (Gaigg 2012 for review). Blunted arousal responses to positive stimuli could reduce attentional engagement, impacting encoding and subsequent memory (Kensinger and Ford 2020; Hamann 2001 for review). Therefore, the diminished perception of emotional intensity of positive images may lead to reduced attentional engagement and ultimately weaker encoding of these stimuli in ASD compared to NT adults. To further investigate whether group differences in arousal were driven by the social content of the images, we conducted an exploratory follow‐up analysis in which all stimuli were post hoc coded as either social or nonsocial (see Supporting Informations) and Group × Social × Valence ANOVAs were conducted for both arousal rating and memory performance (*d'prime*). These follow‐up analyses (i.e., significant Group*Social*Valence interaction) revealed that group differences in arousal were specific to positive images containing social content. No group differences were found for positive nonsocial images or for other valence types. This pattern suggests that attenuated arousal response in ASD could reflect difficulty processing positive social stimuli, rather than positive content more broadly. This finding is again consistent with the aforementioned work on reduced emotional responses specifically to positive social interactions and highlights the importance of accounting for image content, particularly social cues, when evaluating emotional processing differences across groups.

In contrast, memory performance did not show a significant Group*Social*Valence interaction, suggesting that group differences in arousal‐enhanced memory for positive stimuli observed in our original analysis were not fully explained by social content. Together, these results refine our interpretation of group differences in arousal and memory, indicating that social context plays a role in emotional reactivity in ASD, particularly for positive valence stimuli, though its influence on memory appears more limited. ASD arousal‐enhanced reductions for negative stimuli further suggest that memory impairments are not solely driven by subjective arousal ratings at encoding. One possibility is that differences in post‐encoding processes, such as consolidation or retrieval, may contribute to these deficits in ASD, especially for negative emotional memories. Prior studies (Gaigg and Bowler 2008) have found that physiological and subjective arousal do not always align in ASD, concluding that the amygdala may abnormally modulate hippocampal‐based consolidation processes as a function of arousal in ASD due to poor connectivity (e.g., Gaigg and Bowler 2007). Together, findings from the literature and our study suggest that atypical arousal processing across both physiological and behavioral aspects of memory formation may limit the emotional memory benefit in ASD.

Interestingly, memory performance analyses revealed that adults with ASD had reduced arousal‐enhanced memory benefits across both positive and negative stimuli, relative to NT adults. However, age moderated this effect specifically for positive stimuli, supporting our hypothesis that age may exacerbate emotional memory performance differences in ASD. The age‐related decline for positive arousal‐enhanced memory in ASD suggests that emotional memory processes may become increasingly vulnerable with age in this population, particularly for positive stimuli. While recent longitudinal research suggests general cognitive aging trajectories in ASD may parallel those of NT individuals (Torenvliet et al. 2023), our findings support the accelerated aging hypothesis (Geurts and Vissers 2012) as it applies to emotional memory processes reliant on arousal. Adults with ASD may experience earlier or more pronounced declines in emotionally salient memory for positive events, impacting socioemotional functioning.

Finally, our results highlight the role of depression in shaping emotional memory in adults with and without ASD. Although depression did not significantly moderate differences in arousal‐enhanced memory, it was associated with reduced subjective arousal for positive stimuli across both groups. This finding is consistent with cognitive models of depression such as the positive attenuation hypothesis (Clark et al. 1994; Rottenberg et al. 2005; see Carl et al. 2013 for review) which suggests that individuals with depression will have reduced reactivity to positive emotional stimuli. This finding also aligns with meta‐analytic findings that blunted positive emotional reactions in depression are more pronounced, especially on self‐report measures, than differences in negative emotional reactivity (see Bylsma et al. 2008; Bean et al. 2022 for meta‐analyses). Prior research with NT adults has shown that depression is often associated with greater memory impairment of positive more than negative information (James et al. 2021 for meta‐analysis), suggesting that depression's impact may be especially limiting for positive affective processing across the lifespan. Further, while individual differences in depression symptomatology were not directly predictive of memory performance differences between adults with and without ASD, the elevated baseline depression in ASD (Table 1) may partially explain the reduced engagement with positive stimuli observed in this group. The tendency toward blunted positive affect in depression could intensify challenges with attention orienting and encoding of positive stimuli in ASD. Together, our results suggest that while elevated depression in ASD may lead to diminished arousal for positive stimuli, the arousal‐related memory impairments in ASD are primarily driven by ASD‐specific mechanisms rather than comorbid depressive symptoms. This supports the idea that ASD‐related challenges in emotional memory function independently of depression, pointing to unique cognitive mechanisms in ASD that influence memory processes for emotionally salient events.

### 3.1 Limitations

The present study has some limitations. First, all individuals in our study were recruited through an online platform and needed to complete experimental tasks on a computer using an internet‐based interface, which required a certain level of technical proficiency. Therefore, our findings may not extend to individuals with ASD who may have difficulty navigating complex task instructions via digital interfaces, as opposed to simpler online tasks. Another limitation of the current method is that online data collection inhibits us from truly confirming a diagnosis of ASD (with an ADOS‐2 or ADI) as would be done in lab studies, which may affect comparability with clinically confirmed samples. Consistent with other online studies (e.g., Baron‐Cohen et al. 2015; Atherton et al. 2022), we asked ASD participants to provide multiple pieces of information related to diagnosis (i.e., diagnosis given, by whom, when) as well as fill out two self‐report ASD symptom questionnaires in efforts to verify diagnosis. Nonetheless, future studies should aim to replicate these findings in a lab study where clinical diagnostic tools can be administered to confirm ASD diagnoses. Another related limitation is that the autism‐specific self‐report questionnaires (i.e., AQ and SRS‐2) were administered only to participants in the ASD group. While NT participants were screened based on self‐report and Prolific profile data (i.e., no endorsement of ASD diagnosis), it is possible that some individuals in the NT group may have elevated autistic traits that were not disclosed or recognized. Future work would benefit from administering trait‐level measures across both groups to allow for dimensional comparisons and more comprehensive characterization of participant profiles.

Lastly, the online nature of the data collection also prevented us from collecting physiological arousal data (e.g., skin conductance, heart rate). Individuals with ASD may also experience difficulties with metacognition and introspection (Williams and Happé 2010; Grainger et al. 2014; Huggins et al. 2021) which could affect their ability to accurately report on internal states such as arousal and may have contributed to group differences in the pattern of arousal rating. Further, prior research suggests that physiological arousal and self‐reported arousal ratings may not always align in ASD as in NT, potentially capturing different dimensions of the arousal experience (e.g., Gaigg and Bowler 2008). Future studies incorporating both physiological and self‐report measures could determine if the present findings vary based on measurement type, offering a more comprehensive understanding of arousal‐related memory processes in this population.

### 3.2 Conclusion

Our findings make an important contribution to the growing literature on emotional memory in ASD by demonstrating reduced arousal‐enhanced memory benefits across both positive and negative stimuli. These findings suggest that individuals with ASD may have difficulty leveraging arousal to enhance memory, regardless of valence. This is particularly relevant given the well‐documented arousal‐related memory advantages for both positive and negative stimuli in NT populations. Additionally, the age‐related differences in arousal‐enhanced memory benefits for positive stimuli indicate that emotional memory processes in ASD may be especially susceptible to age‐related declines. These findings support the accelerated aging hypothesis in ASD, suggesting that emotional memory for positive stimuli may be particularly vulnerable with age. Finally, while elevated depression symptomatology was associated with reduced arousal ratings for positive stimuli in both groups, it did not impact arousal‐enhanced memory outcomes. Although depression may attenuate positive arousal in both ASD and NT groups, it does not account for the unique arousal‐enhanced memory reductions in ASD. This underscores that diagnosis‐specific factors in ASD play a more significant role in shaping emotional memory processes than comorbid depression alone.

## Author Contributions

**Sidni A. Justus:** conceptualization, methodology, investigation, formal analysis, visualization, writing – original draft, writing – review and editing, supervision. **Emily Hutson:** investigation, visualization, writing – review and editing. **Justin Summe:** investigation, writing – review and editing. **Audrey Duarte:** conceptualization, methodology, writing – review and editing, supervision, resources, project administration, funding acquisition.

## Ethics Statement

All data for this study was collected under an IRB (PI: Dr. Audrey Duarte) approved by Georgia Institute of Technology Ethics Board. Georgia Tech is a former institution of both Dr. Duarte and Dr. Justus.

## Conflicts of Interest

The authors declare no conflicts of interest.

## Supporting information


**Data S1:** Supporting Information.

## Data Availability

Data used in the current study are available at https://osf.io/9wa6c/?view_only=5b977f6d7b524f028d042bc24831df31.
