# Supplementary material for: Emotional Arousal‐Induced Episodic Memory Benefits Are Attenuated in Autism Spectrum Disorders, Especially in Older Age
Source: Autism Res. 2025 Jul 14;18(9):1817–29. doi: 10.1002/aur.70083 (PMC12329672; doi:10.1002/aur.70083)
Supplement: Supplementary file 1 — Data S1: Supporting Information. [file AUR-18-1817-s001.docx]

**Supplemental Materials**

**Method**

**Health Questionnaire Items Related to Autism Diagnosis**

Do you have or have you ever been diagnosed with any of the following:

Autism Spectrum Disorders or related disorders (e.g., Asperger’s)? - *Y/N*

What was the clinical diagnosis you were given?(e.g., Autism, Autism Spectrum Disorder, Asperger's, Rhett's) - *Open Ended*

When? (year) - *Open Ended*

By whom? (e.g., psychiatrist) - *Open Ended*

How old were you at time of diagnosis? - *Open Ended*

Have you received any treatment or intervention related to this diagnosis? If so, please elaborate on type/activity and when? - *Open Ended*

**Post-hoc Exploratory Analysis: Social Content of Stimuli**

To evaluate whether group differences in arousal were driven by the social nature of the stimuli, particularly for positive images, we conducted exploratory follow-up analyses. The second and third authors independently coded each image used in the study as either social (e.g., including people, eye contact, social interaction) or nonsocial. Cohen’s Kappa indicated strong agreement, κ = .82, *p* < .001, with discrepancies resolved by the first author. Coding results are summarized in **Supplementary Table 1.** We next ran separate 2 (Group: NT, ASD), x 2 (Social: Social, Nonsocial) x 3 (Valence: Positive, Neutral, Negative) repeated measures ANOVAs on arousal ratings and memory performance (*d’prime).* Greenhouse-Geisser corrections were applied as needed when the assumption of sphericity was violated.

**Supplementary Table 1.** *Social vs. Nonsocial counts for stimuli.*

| **Valence** | **Old/New Status at Retrieval** | **Total # Social Images** | **Total # Nonsocial Images** | **Image Type Totals** |
| --- | --- | --- | --- | --- |
| Pos | New | 12 | 32 | 44 |
| Neu | New | 15 | 29 | 44 |
| Neg | New | 8 | 36 | 44 |
| Pos | Old | 33 | 55 | 88 |
| Neu | Old | 10 | 78 | 88 |
| Neg | Old | 23 | 65 | 88 |

**Results**

Average group subjective arousal ratings and memory performance (*d’prime*) for each social and valence category are shown in **Supplementary Table 2.**

**Supplementary Table 2.** *Group subjective arousal ratings and memory discriminability (d’) as mean (standard deviation) by valence, group, and social content.*

| **Measure** | **Social Content** | **Group** | **Positive** | **Neutral** | **Negative** |
| --- | --- | --- | --- | --- | --- |
| **Arousal Rating** | Social | NT | 1.87 (.58) | 1.46 (.45) | 2.43 (.51) |
|  |  | ASD | 1.75 (.53) | 1.43 (.43) | 2.52 (.62) |
|  | Nonsocial | NT | 2.02 (.56) | 1.50 (.39) | 2.40 (.44) |
|  |  | ASD | 1.95 (.55) | 1.53 (.40) | 2.44 (.53) |
| **Overall *d*′** | Social | NT | 1.55 (.71) | 1.43 (.73) | 1.83 (.81) |
|  |  | ASD | 1.56 (.74) | 1.23 (.76) | 1.65 (.81) |
|  | Nonsocial | NT | 1.47 (.66) | 1.60 (.79) | 1.86 (.71) |
|  |  | ASD | 1.35 (.64) | 1.45 (.74) | 1.69 (.70) |

*Note.* Entries are in the format: *Mean* (*SD*). *d′* = *z(*hit rate) – *z*(false alarm rate).

**Arousal**

Results from the Group (NT, ASD) x Social (Social, Nonsocial) x Valence (Positive, Neutral, Negative) ANOVA revealed a main effect of Valence [*F*(1.71, 554.09) = 889.31, *p* < .001, *η_p_*^2^ = .73], but no main effect of Group [*F*(1, 324) = 0.06, *p* = .809, *η_p_*^2^ = .00], replicating patterns observed in the primary analysis. Similarly, a Group*Valence interaction was replicated, [*F*(1.71, 554.08) = 6.30, *p*  = .003, *η_p_*^2^ = .02]. A main effect of Social, [*F*(1, 324) = 29.86, *p* < .001, *η_p_*^2^ = .08] and Social*Valence interaction, [*F*(1.89, 612.05) = 101.99, *p* < .001, *η_p_*^2^ = .24] were revealed, but the Social*Group interaction was not significant, [*F*(1, 324) = 0.59, *p* = .443, *η_p_*^2^ = .00]. Finally, a significant Group*Social*Valence interaction was revealed, [*F*(1.89, 612.05) = 5.51, *p*  = .005, *η_p_*^2^ = .02]. Follow-up Group x Valence ANOVAs were conducted separately for social and nonsocial images to explore the three-way interaction. For Social images, results revealed a main effect of Valence, [*F*(1.66, 539.23) = 815.10, *p* < .001, *η_p_*^2^ = .72], and a significant Group*Valence interaction, [*F*(1.66, 539.23) = 8.23, *p* < .001, *η_p_*^2^ = .03]. There was no main effect of Group, [*F*(1, 324) = 0.17, *p* = .685, *η_p_*^2^ = .00]. Independent samples t-tests indicated that adults with ASD rated positive social images as less arousing than did NT adults, *t*(324) = 1.99, *p* = .024, Cohen’s *d* = 0.22. No significant group differences were revealed for neutral or negative social images, (*t*(324)’s < 1.41, *p’s* > .08*,* Cohen’s *d*’s < .16).

For Nonsocial images, results again revealed a main effect of Valence, [*F*(1.83, 593.31) = 776.86, *p* < .001, *η_p_*^2^ = .71], and a significant Group*Valence interaction, [*F*(1.83, 593.31) = 3.60, *p* = .032, *η_p_*^2^ = .01]. There again was no main effect of Group, [*F*(1, 324) = 0.00, *p* = .956, *η_p_*^2^ = .00]. However, follow-up Independent samples t-tests revealed no significant group differences in arousal ratings for positive, neutral, or negative nonsocial images, (*t*(324)’s < 1.17, *p’s* > .12*,* Cohen’s *d*’s < .13). These findings suggest that the three-way interaction was driven by differences in arousal to positive social images, with adults with ASD showing blunted arousal compared to NT adults. Although a significant Group*Valence interaction emerged for nonsocial images, no individual comparisons reached significance, which may reflect subtle group differences not captured by pairwise contrasts.

***Memory Performance***

Results from the Group (NT, ASD) x Social (Social, Nonsocial) x Valence (Positive, Neutral, Negative) ANOVA revealed a main effect of Valence [*F*(2, 556) = 88.92, *p* < .001, *η_p_*^2^ = .24], but no main effect of Group [*F*(1, 278) = 3.42, *p* = .065, *η_p_*^2^ = .01], and no Group*Valence interaction [*F*(2, 278) = 3.01, *p* = .050, *η_p_*^2^ = .01], replicating patterns observed in the primary analysis. A significant Social*Valence interaction was revealed, [*F*(2, 556) = 23.05, *p* < .001, *η_p_*^2^ = .08]. Follow-up repeated-measures ANOVAs were conducted separately for social and nonsocial images to explore this interaction. In both cases, a significant main effect of Valence was revealed, [Social: *F*(2, 558) = 47.01, *p*  < .001, *η_p_*^2^ = .14; Nonsocial: *F*(2, 646) = 81.08, *p*  < .001, *η_p_*^2^ = .20], indicating that memory performance varied across valence levels regardless of social content. Follow-up Paired Samples t-tests revealed that memory performance was higher for positive Social (*M* = 1.46, *SD* = 0.74) than Nonsocial (*M =* 1.35, *SD* = 0.64) images, [*t*(323) = 3.87, *p* < .001, Cohen’s *d* = .22]. In contrast, participants demonstrated higher memory performance for neutral Nonsocial (*M =* 1.52, *SD* = 0.77) than Social (*M* = 1.32, *SD* = 0.75) images, [*t*(280) = -5.21, *p* < .001, Cohen’s *d* = .31]. There was no significant difference between negative Social (*M* = 1.66, *SD* = 0.83) and Nonsocial (*M =* 1.68, *SD* = 0.74), images [*t*(323) = -0.60, *p* = .550, Cohen’s *d* = .03]. There were no other significant effects [all *F*’s < 1.98, *p*’s > .139, *η_p_*^2^ ‘s < .01].

Collectively, these findings suggest that memory performance was influenced by the social content of images, particularly for positive and neutral stimuli. However, the absence of group differences or interactions indicates that social content alone does not explain the group-based differences in arousal-enhanced memory observed in the primary analyses. Finally, because the number of social and nonsocial stimuli was not balanced across valence categories (see **Supplemental Table 1**), results should be interpreted with caution. Stimuli for this study were chosen from the IAPS and NAPS image banks without initial selection based on social content.
